# Concepts in Multifactorial Etiology of Developmental Disorders: Gene-Gene and Gene-Environment Interactions in Holoprosencephaly

**DOI:** 10.3389/fcell.2021.795194

**Published:** 2021-12-22

**Authors:** Hsiao-Fan Lo, Mingi Hong, Robert S. Krauss

**Affiliations:** Department of Cell, Developmental, and Regenerative Biology, Icahn School of Medicine at Mount Sinai, New York, NY, United States

**Keywords:** birth defect, holoprosencephaly, hedgehog signaling, teratogen, fetal alcohol, genetics, epidemiology

## Abstract

Many common developmental disorders are thought to arise from a complex set of genetic and environmental risk factors. These factors interact with each other to affect the strength and duration of key developmental signaling pathways, thereby increasing the possibility that they fail to achieve the thresholds required for normal embryonic patterning. One such disorder, holoprosencephaly (HPE), serves as a useful model system in understanding various forms of multifactorial etiology. Genomic analysis of HPE cases, epidemiology, and mechanistic studies of animal models have illuminated multiple potential ways that risk factors interact to produce adverse developmental outcomes. Among these are: 1) interactions between driver and modifier genes; 2) oligogenic inheritance, wherein each parent provides predisposing variants in one or multiple distinct loci; 3) interactions between genetic susceptibilities and environmental risk factors that may be insufficient on their own; and 4) interactions of multiple genetic variants with multiple non-genetic risk factors. These studies combine to provide concepts that illuminate HPE and are also applicable to additional disorders with complex etiology, including neural tube defects, congenital heart defects, and oro-facial clefting.

## Introduction

**FIGURE 1:**
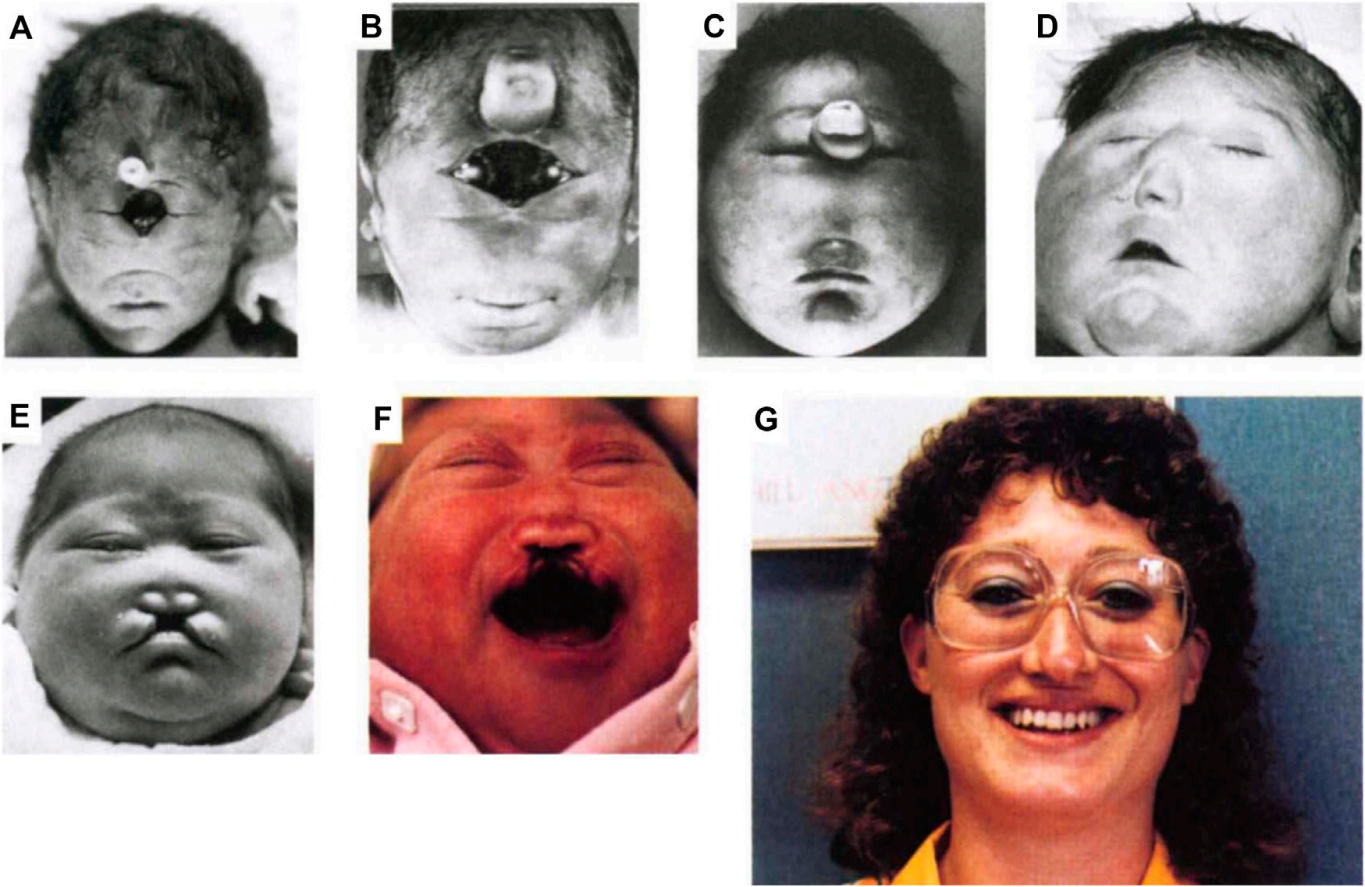
Spectrum of HPE phenotypes. A spectrum of facial phenotypes in patients with HPE, including cyclopia with a proboscis **(A)**, undivided eye field with proboscis **(B)**, proboscis between separated eyes **(C)**, closely spaced eyes (hypotelorism) and single-nostril nose **(D)**, hypotelorism with midfacial hypoplasia and midline cleft lip **(E)**, hypotelorism, absence of nasal bones, and midline cleft lip **(F)**, and solitary median maxillary central incisor **(G)**. Reprinted with permission of Springer Nature (Roessler et al., 1996).

Holoprosencephaly (HPE) is a very common developmental disorder defined as a failure in midline patterning of the forebrain and/or midface (Muenke and Beachy, 2001; Tekendo-Ngongang et al., 2020). It is usually stated that HPE arises approximately once per 250 conceptuses, but a recent study suggested that this figure may be as high as once per 32 conceptuses (Shiota and Yamada, 2010; Shiota, 2021). Due to *in utero* lethality, live birth frequency is only ∼1 in 10,000 (Leoncini et al., 2008). An unbroken continuum of HPE phenotypes (sometimes called the HPE spectrum) is broadly classified into three categories based on the degree of midline cleavage of the forebrain (Muenke and Beachy, 2001; Tekendo-Ngongang et al., 2020). Alobar HPE, the most severe form, is characterized by complete failure to partition the forebrain into left and right hemispheres, resulting in a single, centrally-located ventricle. Semilobar and lobar HPE are progressively less severe forms and display partial, or mostly complete, forebrain cleavage, respectively. HPE-associated midline anomalies of the face include cyclopia, single nostril, midfacial midline clefting, hypotelorism (i.e., very close set eyes), and solitary median maxillary central incisor (Figure 1). Mild facial midline abnormalities may occur without clinically obvious brain malformations and are called HPE microforms (Tekendo-Ngongang et al., 2020). A variant HPE subtype, middle interhemispheric (MIH) HPE, is relatively rare and differs in its developmental origins from the classical forms (Monuki, 2007; Tekendo-Ngongang et al., 2020), which are all thought to share a similar etiology but vary in expressivity.

Development of the forebrain is a complex, multistep process [for detailed reviews, see (Wilson and Houart, 2004; Grinblat and Lipinski, 2019)]. Briefly, neuroectodermal cells at the rostral end of the neuraxis develop into the anterior neural plate, followed by dorsoventral patterning. The forebrain in turn provides signals that pattern the face. It has long been said for HPE that, “the face predicts the brain”, an aphorism that holds true in about 80% of cases (DeMyer, 1964; Krauss, 2007). HPE is generally associated with defects in ventral patterning, a process regulated by morphogenetic signals that begin with Nodal pathway signaling during gastrulation, followed by Hedgehog (HH) and FGF pathway signaling in the ventral midline of the developing forebrain and, ultimately, facial primordia (Wilson and Houart, 2004; Marcucio et al., 2015; Grinblat and Lipinski, 2019). Mutations in genes encoding components and regulators of these pathways are found in HPE. Partitioning of the forebrain into hemispheres initiates dorsally, and it is not clear how defects in ventral patterning perturb this process in classical HPE. In contrast, MIH HPE appears to arise as a consequence of defects in dorsal patterning, and the characteristic craniofacial abnormalities associated with classical HPE are observed infrequently in this form of HPE (Monuki, 2007; Fernandes and Hébert, 2008).

The wide spectrum of defects that characterize classical forms of HPE likely arise from alterations in both signaling pathway levels and timing. Sonic HH (SHH) can function as a morphogen, specifying distinct outcomes for cells within a target field in a concentration-dependent manner; failures to reach signaling output thresholds required for specific development patterning events may help dictate a spectrum of HPE phenotypes (Young et al., 2010; Sagner and Briscoe, 2019). The developmental stage at which suboptimal signaling by these pathways occurs also plays a significant role in the expressivity of HPE outcomes, with earlier deficits resulting in more severe phenotypes, and progressively later deficits yielding progressively less severe phenotypes (Cordero et al., 2004; Krauss, 2007; Marcucio et al., 2015).

HPE occurs most often as part of a syndrome; some of these syndromes are associated with specific chromosomal aberrations, including various trisomies, structural chromosomal abnormalities, and pathogenic copy number variations (CNVs) (Kruszka and Muenke, 2018). Isolated HPE (i.e., HPE not associated with gross chromosomal aberrations or as a feature of a syndrome) accounts for approximately ∼25% of cases but ∼75% of patients, and occurs both in pedigrees and sporadically (Roessler et al., 2018a; Tekendo-Ngongang et al., 2020). Mutations at known gene loci have been identified in <30% of individuals with isolated HPE (Kim et al., 2018; Tekendo-Ngongang et al., 2020). Clinical presentation of HPE is highly variable, even in pedigrees, and many mutation carriers in these families have no obvious clinical manifestation (Lacbawan et al., 2009; Solomon et al., 2012; Stokes et al., 2018). It appears that a complex interplay of multiple genetic and/or environmental influences underlies a substantial fraction of isolated HPE cases (Hong and Krauss, 2018; Beames and Lipinski, 2020).

One may ask, why study HPE? The developmental events that go awry happen in the third and fourth weeks of human gestation, before many women know they are pregnant (Hong and Krauss, 2018; Tekendo-Ngongang et al., 2020). Reversing the causative developmental patterning defects after they have occurred is not possible, so successful therapeutic intervention is unlikely. Moreover, the vast majority of holoprosencephalic fetuses succumb *in utero*, often in the first trimester (Shiota and Yamada, 2010; Shiota, 2021). A superficial focus on the relatively low live birth frequency, however, ignores the difficulties facing surviving patients and their families, as well as the pain to families experiencing pregnancy loss. There are many additional important reasons to study HPE. First, understanding the etiology of HPE may aid in its prevention. The prime example of such a success is maternal dietary supplementation of folic acid, which reduces the risk of neural tube defects, another developmental disorder associated with errors in early patterning and high rates of prenatal mortality (Finnell et al., 2021). Second, HPE serves as an ideal model of a developmental disorder with complex, multifactorial etiology. Recent genomic analyses and epidemiological studies promise insight into this phenomenon. Animal models accurately mimic this situation and allow both experimental validation of observations made in human populations and testing of ideas that may provide new leads in human studies. Importantly, multifactorial etiology is likely to apply to many developmental disorders. HPE is well positioned to serve as a model system in which broadly applicable concepts are established. Here we review the evidence for multifactorial etiology in HPE in humans and how animal models contribute to our understanding of this phenomenon.

## Gene-Gene Interactions in HPE

The three most frequently mutated genes in isolated HPE are *SHH*, *ZIC2*, and *SIX3*, but across many studies none of them accounts for more than 10% of total cases (Tekendo-Ngongang et al., 2020). Mutations in *FGF8* and *FGFR1* are also relatively frequently observed in isolated HPE (>2% of cases), but they are also implicated in syndromes that include HPE (e.g., Kallman and Hartsfield syndromes) (Dubourg et al., 2016; Roessler et al., 2018a). Interestingly, most of these factors interact during rostroventral midline patterning; SHH and SIX3 regulate each other’s expression, and the FGF and HH signaling pathways cross-regulate each other’ activities (Wilson and Houart, 2004; Geng et al., 2008; Grinblat and Lipinski, 2019). Mutations in many other genes (mainly encoding components and regulators of HH signaling) have been identified, but these are considered “minor” or rare HPE loci (≤1% of cases for individual genes) (Bae et al., 2011; Dubourg et al., 2016; Roessler et al., 2018a; Roessler et al., 2018b; Kruszka et al., 2019a; Kruszka et al., 2019b; Tekendo-Ngongang et al., 2019). Virtually all these mutations are heterozygous and, where tested, are generally loss-of-function variants. Autosomal recessive mutations in HPE have been documented, but are very rare. Examples include mutations in *HHAT* (encoding Hedgehog acyltransferase), *PLCH1* (encoding phospholipase C eta-1), and *STIL* [encoding a factor required for maintenance of primary cilia, a subcellular structure critical for HH signaling (Kakar et al., 2015; Mouden et al., 2015; Drissi et al., 2021; Pande et al., 2021)].

### Driver and Modifier Genes in HPE

Heterozygous mutations in the most frequently involved loci are viewed as “drivers” of HPE, in that the alleles are of low frequency in the population, their functions fit the known developmental biology of HPE, and they are generally accepted to be essential to the phenotype. But are they actually sufficient to induce the full range of phenotypes seen in HPE patients? For *SHH* and *SIX3*, the answer to this question is likely, “no”. Only 10 and 14% of *SHH* and *SIX3* mutations occur *de novo*, respectively (Lacbawan et al., 2009; Solomon et al., 2012). Additionally, large HPE pedigrees exist wherein many individuals across generations have mutations in either *SHH* or *SIX3*, with up to ∼30% of carriers lacking obvious clinical manifestation and the rest displaying a full spectrum of phenotypes (Lacbawan et al., 2009; Solomon et al., 2012). Furthermore, even in sporadic HPE cases, the majority of *SHH* and *SIX3* mutations are inherited from unaffected, or very mildly affected, parents (Lacbawan et al., 2009; Solomon et al., 2012). In contrast, *ZIC2* heterozygosity may be sufficient. More than 70% of *ZIC2* mutations arise *de novo*, and large pedigrees have not been observed (Solomon et al., 2010). Interestingly, *ZIC2*-associated HPE is phenotypically distinct from that associated with *SHH* and *SIX3*, in that it lacks the classical facial midline features of the latter (Solomon et al., 2010).

A statistical evaluation of these results led to an “autosomal dominant mutation plus modifier” model, in which the penetrance and expressivity of heterozygous mutations in *SHH* or *SIX3* (or other, rarer HPE genes) is determined by additional risk factors—genetic, environmental, or both—acting as modifiers (Roessler et al., 2012). Initial evidence for the existence of HPE modifier genes came from studies with mice. Germline mutation of HPE driver genes in mice also produces HPE, but for reasons that are still not fully clear, mice require homozygous mutations for phenotypic manifestation (i.e., HPE is autosomal recessive in mice) (Hong and Krauss, 2018). Even in the homozygous mutant state, the penetrance and expressivity of HPE for some of these genes is highly dependent on the genetic background of the mice. C57BL/6 mice are a more sensitive strain than are various 129 substrains for HPE associated with null mutation of *Six3*, as well as of the rare HPE genes, *Cdon*, and *Gli2* (Zhang et al., 2006; Geng et al., 2008; Heyne et al., 2016). Therefore, the genetic background differences in these strains function to modify the phenotypic outcome of the same mutation. The gene loci responsible for HPE sensitivity *vs.* resistance in these various inbred lines have not been identified. In fact, differential strain sensitivity to many mutations has long been recognized, but few such modifiers have been found. Alternative approaches have therefore been pursued. Mecklenburg et al. recently reported that mutation of *Lrp2* (which encodes an auxiliary HH receptor) produces HPE in C57BL/6N mice but not in FVB/N mice (Mecklenburg et al., 2021). The authors generated transcriptomes from embryonic forebrains of wild type and *Lrp2* mutant mice of both backgrounds and of F1 mice (which, like FVB/N mice, are resistant to *Lrp2* mutation). Comparative analysis of these datasets uncovered differentially expressed genes encoding previously unidentified regulators of HH signaling (Mecklenburg et al., 2021). These genes may in turn be candidate modifiers in human HPE.

Identification of HPE modifier genes has been challenging, and few are known. A classical modifier gene with high explanatory power for the variability of HPE phenotypes would be anticipated to have alleles with a frequency higher than the live birth rate and be enriched in HPE cases, relative to the general population. Furthermore, at least some HPE patients who carry the putative modifier allele should also have heterozygous mutations in known HPE driver genes, providing a genetic substrate on which the modifier acts. Finally, such modifier genes should have a biological function consistent with a role in rostroventral midline patterning (though such functions may be unknown at the time of the modifier’s discovery). Only one such modifier has been identified: *BOC*, which encodes a HH coreceptor (Petrov et al., 2017). In studying *BOC* missense variants present in HPE patients, two alleles were identified in cases that also had mutations in either *ZIC2* or *TGIF1* (a *bone fide* HPE gene) (Hong et al., 2017). One *BOC* variation had a minor allele frequency of 0.0017, higher than the HPE live birth frequency of 1:10,000. These alleles were then demonstrated to have a loss of function in *in vitro* HH signaling assays (Hong et al., 2017). *BOC* missense alleles are not commonly found in HPE, however, suggesting they are relatively low frequency participants in this disorder. Nevertheless, there are hundreds of *BOC* missense mutations listed in gnomAD, and their potential roles are as yet unclear.

Strengthening the conclusion that *BOC* is a human HPE modifier gene is that its murine counterpart acts as a true silent HPE modifier gene in mice. *Boc*-null mice do not have HPE, but removal of *Boc* from *Cdon* mutant mice enhances HPE associated with the latter, on both C57BL/6 and 129 backgrounds (Zhang et al., 2011). However, BOC regulates HH-dependent craniofacial patterning in complex ways. Although *Boc*-null mice do not display defects in craniofacial development on any studied genetic background, *Boc* mutations interact differentially with mutations of another HH coreceptor, GAS1, dependent on the genetic background. *Boc* mutation enhances craniofacial midline defects of *Gas1* mutants on a mixed genetic background, whereas it partially rescues such defects in *Gas1* mutants on a C57BL/6 background (Seppala et al., 2014; Echevarría-Andino and Allen, 2020). BOC, as well as its paralog CDON, can therefore function as both a positive and negative regulator of HH-dependent patterning in various model organisms (Gallardo and Bovolenta, 2018). Significantly, a *BOC* variant identified in an HPE patient displayed a HH ligand-dependent, *gain*-of-function phenotype in in vitro assays, opposite what would be predicted for an allele that promoted HPE (Hong et al., 2017). This variant is unique to the genome databases and may represent a rare HPE suppressor allele that dampened clinical phenotypes, allowing patient survival and the ability to be analyzed. These results suggest that HPE modifiers may be very complex in function.

### Oligogenic Inheritance

A view of gene-gene interactions in HPE etiology that is complementary to an “autosomal dominant driver mutation-plus-modifier” model is a more generalized oligogenic inheritance model; i.e., HPE can arise from a combination of multiple inherited mutations, without necessarily involving a strict hierarchy of driver and modifier genes (Dubourg et al., 2018). Until relatively recently, targeted sequencing for mutations in four known HPE genes (*SHH*, *ZIC2*, *SIX3*, and *TGIF1*) was the standard applied to new cases, and very few cases presented with variants in more than one gene. As the list of potential HPE genes grew, and whole exome sequencing (WES) became more affordable, it became possible to screen many individuals in families for variants in many genes at once. Kim et al. applied this approach to 26 families in which asymptomatic or mildly affected parents had children with HPE, ranging from alobar to microform HPE (Kim et al., 2018). A prioritization strategy that included bioinformatic analyses, expression analyses, and mouse knockout phenotypes led to a focus on 180 genes and, in turn, identification of oligogenic inheritance in 10 of the 26 families. HPE cases had between two and five variants from the list of 180 genes and always inherited at least one variant from each parent. Mutations in *SHH*, a classic driver gene, and in *BOC*, the sole known gene that can be viewed as a pure modifier, were identified in multiple cases, including one that had variants in both genes (Kim et al., 2018). This study also identified genes not previously implicated in human HPE, but for which mouse studies suggested a potential role, including *COL2A1*, and *NDST1*. *COL2A1* encodes a collagen isoform and *NDST1* encodes a heparan sulfate-modifying enzyme; these factors act extracellularly to regulate HH signaling in forebrain and craniofacial development (Grobe et al., 2005; Leung et al., 2010). Additionally, variants in several genes encoding components of primary cilia were identified (Kim et al., 2018). Primary cilia are the cellular site of HH signaling to GLI transcription factors (Gigante and Caspary, 2020). Finally, some recurrent oligogenic events were observed, including two families with variants in *BOC* and *SCUBE2*. *SCUBE2* encodes a secreted chaperone for HH ligands, which interacts directly with the HH coreceptor BOC (Petrov et al., 2017).

Statistical analyses demonstrated that oligogenic inheritance in the selected candidate genes occurred much more frequently in HPE cases than controls; nevertheless, it will be valuable to follow up these observations with experimental approaches. First, functional analyses on these alleles can be performed to assess whether they are indeed loss-of-function variants. Second, it will be interesting to generate mouse models to further test the specificity of these genetic interactions. For example, CNVs and a single example of a nucleotide variant in the Notch ligand *DLL1* were identified in HPE patients, suggested a previously unknown role for the Notch pathway in rostroventral midline patterning (Dupé et al., 2010). Subsequent studies with mice demonstrated a genetic interaction between Notch and HH signaling in development of the pituitary gland, a structure commonly affected in HPE and midline disorders (Hamdi-Rozé et al., 2020).

There is much overlap between the autosomal dominant mutation-plus-modifier model and oligogenic inheritance model. In the latter, genes considered drivers (e.g., *SHH*) were sometimes found, suggesting that some of the additional variant genes present in specific cases may have provided a modifier function to heterozygous loss of a more powerful driver mutation. Additionally, the presence of as many as five variants in some cases of oligogenic inheritance logically suggests that not all variants contribute equally strongly towards the ultimate phenotype. In both models, a combination of genetic variants interact to elevate the likelihood of a defect in rostroventral midline patterning. Furthermore, both models offer potential explanatory power for the incomplete penetrance and variable expressivity that is characteristic of HPE.

## Gene-Environment Interactions in HPE

Multigenic interactions provide an appealing explanation for many of the complexities associated with HPE, and such analyses should continue to bear fruit. However, mutations have been identified in only a minority of isolated HPE cases, even in WES studies that led to discovery of new HPE genes (Kruszka et al., 2019a; Kruszka et al., 2019b; Tekendo-Ngongang et al., 2019). The fraction of cases with an identifiable genetic component is sure to rise as whole genome sequencing and yet more sophisticated bioinformatic analyses are applied to HPE. However, HPE has long been associated with teratogenic causes also, and it is possible that in some individual cases, mutations and/or genetic predispositions are irrelevant or only minor etiological factors.

The archetypal HH pathway inhibitor cyclopamine was discovered as the major teratogen in corn lilies, which when eaten by pregnant ewes caused cyclopia in their offspring (Chen, 2016). This tour de force of agricultural and scientific discovery demonstrated that *in utero* exposure to a chemical inhibitor of HH signaling at a sensitive period of development is sufficient to induce the most severe form of HPE. Although people do not consume corn lilies, exposure to specific teratogens, or to combinations of non-genetic risk factors, may therefore contribute to human HPE, potentially working with the types of genetic predisposition described in the previous section.

Several epidemiological studies of HPE have been performed, but conclusions were limited by both the number of cases available (generally live births) and the likelihood of incomplete reporting on exposures that occurred during the very early sensitive period for HPE (the third to fourth weeks after conception) (Linn et al., 1983). Maternal pregestational diabetes, which is implicated in several structural birth defects, has reproducibly been associated with elevated HPE risk in these studies (Miller et al., 2010; Summers et al., 2018; Addissie et al., 2021). Prenatal alcohol exposure (PAE) has also been implicated as a risk factor for HPE, in some but not all studies (Croen et al., 2000; Miller et al., 2010; Abe et al., 2018; Summers et al., 2018; Addissie et al., 2021). This may be partly related to how much detail is obtained from questionnaires; one recent case-control study did not find an association between HPE and alcohol consumption *vs.* non-consumption, but increasing amounts of alcohol consumed correlated with higher HPE risk, suggesting a possible threshold effect and dose-responsive outcomes (Addissie et al., 2021).

### Prenatal Alcohol Exposure

PAE is an acknowledged human teratogen and the cause of fetal alcohol spectrum disorders (FASD), including fetal alcohol syndrome (FAS) (Hoyme et al., 2005; Hoyme et al., 2016). An overlap between FASD and mild forms of HPE has been noted by clinicians and laboratory scientists since the 1980s (Sulik and Johnston, 1982; Webster et al., 1983; Neri et al., 1988). Defects of the midfacial midline are commonly seen in FAS, including smooth philtrum, and hypoplastic midface (Hoyme et al., 2005; Hoyme et al., 2016). Midline CNS structures affected in HPE, such as the corpus collosum, are also disproportionately affected in FAS (Coulter et al., 1993; Johnson et al., 1996; Bookstein et al., 2002; Suttie et al., 2018). Two recent papers from leading FASD clinicians reported that reduced interpupillary distance and its severe form, hypotelorism (which are midline patterning defects common in HPE) are useful diagnostic criteria in FASD (Bandoli et al., 2020; Gomez et al., 2020). HPE phenotypes are restricted to the midline, while FASD phenotypes are not; however, the midline defects that define milder HPE are a common feature of FASD, leading numerous investigators to conclude that related mechanisms account for these similarities. As mild HPE phenotypes are a common feature of FASD, this scenario can be viewed as analogous to HPE being a feature of specific genetic syndromes.

Studies with mice offer strong support for this point of view. In 1981, Sulik and colleagues developed a mouse model of FASD with C57BL/6 mice, and it was quickly noted that HPE-like phenotypes were among those observed (Sulik et al., 1981; Sulik and Johnston, 1982). As noted above, C57BL/6 mice are sensitive to mutation-induced HPE, and HPE is observed in ∼20% of the mice subjected to this PAE protocol (Aoto et al., 2008). Furthermore, PAE-induced HPE is enhanced in C57BL/6 mice heterozygous for *Shh* or *Gli2*, thereby demonstrating a gene-environment interaction (Kietzman et al., 2014). 129S6 mice, which are much more resistant to mutation-induced HPE than C57BL/6 mice, are also resistant to PAE-induced HPE, and other craniofacial phenotypes (Downing et al., 2009; Hong and Krauss, 2012). 129S6 mice with a mutation in the HH coreceptor CDON have a subthreshold deficit in HH signaling and are sensitive to HPE induced by “second hits”, genetic or environmental (Hong and Krauss, 2018). PAE in these mice produced a complete spectrum of HPE phenotypes with high penetrance and high fidelity to human HPE (Hong and Krauss, 2012). PAE therefore induces HPE in mice that are genetically sensitive due to: 1) strain background modifiers; 2) the presence of true predisposing mutations; or 3) both. These results suggest that, in humans, PAE may function as an environmental modifier of HPE.

### Δ9-Tetrahydrocannabinol

In 2015, Khaliullina et al. demonstrated that endocannabinoids, a class of endogenous fatty acids/alcohols, inhibited HH signaling in developing fruit flies, and cultured mouse cells (Khaliullina et al., 2015). Additionally, phytocannabinoids, the active ingredients in cannabis [e.g., Δ9-tetrahydrocannabinol (THC) and cannabidiol], also inhibited HH signaling in cultured cells. This raised the possibility that *in utero* exposure to phytocannabinoids might be teratogenic, perhaps in concert with genetic predisposition. This hypothesis was tested with *Cdon* mutant mice, already proven to be valuable for testing the effects of HPE modifiers. THC inhibited HH signaling during development of 129S6 *Cdon* mutant embryos, resulting in two hallmark HH loss of function phenotypes: mild HPE and ventral neural tube patterning defects (Lo et al., 2021). THC produced these effects in *Cdon* mutants but not wild type mice, indicating it acted as a conditional teratogen, dependent on a complementary but insufficient genetic defect. THC acts as a direct, albeit relatively weak, inhibitor of the essential HH signal transducer Smoothened (SMO), the same target as the more potent teratogen cyclopamine (Chen et al., 2002; Lo et al., 2021). Interestingly, THC also exacerbated developmental defects induced by PAE in C57BL/6 mice (Fish et al., 2019). THC is therefore a potential environmental risk factor for HPE and other developmental disorders linked to HH signaling deficiency. Recent epidemiological studies have correlated increased cannabis usage with specific structural birth defects (Reece and Hulse, 2019; Reece and Hulse, 2020). Additional work is needed to address the possibility that cannabis usage during early pregnancy is teratogenic to humans, and whether individuals with genetic predisposition may be at elevated risk.

### Piperonyl Butoxide

Studies with agricultural and experimental animals demonstrate that SMO inhibitors are HPE-inducing teratogens. SMO is a seven-pass transmembrane protein of the G protein-coupled receptor superfamily. It has multiple binding modalities for small molecules, and many SMO agonists and antagonists have been identified (Sharpe et al., 2015). There are thousands of synthetic compounds present in the environment and it is possible that among them exist some which inhibit SMO and could be HPE risk factors. Wang et al. used a high-content cell culture assay to test a library of more than 1,400 environmental toxicants for SMO antagonist activity (Wang et al., 2012). One SMO inhibitor was identified: piperonyl butoxide (PBO), a pesticide synergist in wide use and among the top 10 chemicals detected in indoor dust (Rivera-González et al., 2021). In utero exposure of C57BL/6 mice to PBO dose-dependently produced forebrain and facial phenotypes characteristic of HPE (Everson et al., 2019). Furthermore, C57BL/6 *Shh* ^*+/−*^ mice were sensitized to lower doses of PBO (Everson et al., 2019). Importantly, a recent case-control study provided evidence that maternal exposure to pesticides during pregnancy elevated the risk of HPE (Addissie et al., 2020). Follow-up studies with larger cohorts are clearly warranted.

### Future Directions in Studying Gene-Environment Interactions in HPE

The structures of small molecule SMO antagonists are diverse, and they vary in potency. High-resolution structures of SMO alone or bound by natural or synthetic inhibitors have been derived (Sharpe et al., 2015; Kowatsch et al., 2019; Qi and Li, 2020), and it may be possible to combine this information with modeling studies to identify potential SMO inhibitors among the enormous number of unregulated chemicals present in the environment. Although SMO inhibitors are clearly a concern as potential HPE risk factors, SMO inhibition is not the sole mechanism whereby chemical compounds may raise the risk of HPE. First, HH signaling is subject to small molecule inhibition at multiple steps, and other components of the pathway could also be targets of potential teratogens (McMillan and Matsui, 2012). Second, other pathways are also relevant to HPE. For example, the Nodal pathway lies developmentally upstream of the HH pathway in rostroventral midline patterning. Ethanol’s major HPE-inducing teratogenic effect in mice is likely *via* inhibition of Nodal signaling, with effects on HH signaling occurring as an indirect consequence (Hong et al., 2020). Although the direct target of ethanol’s inhibitory effects on Nodal signaling are as yet unknown, it rapidly induces an inhibitory pattern of phosphorylation in SMAD2, the pathway-responsive transcription factor (Hong et al., 2020). In zebrafish, ethanol also inhibits anterior migration of the prechordal plate, a key structure induced by Nodal signaling and which secretes SHH (Blader and Strahle, 1998).

Recent epidemiological studies have revealed additional potential environmental risk factors for HPE. Polycyclic aromatic hydrocarbons (PAHs) are produced by incomplete combustion of naturally occurring organic compounds and are present at high levels in specific work environments, as well as in cigarette smoke and charred meats. A 2020 study indicated that maternal occupational exposure to PAHs elevated the risk for HPE and selected other defects of the face and CNS (Santiago-Colón et al., 2020). A second recent study by Addissie et al. identified pregnancy-associated risk with exposure to consumer products such as bleach, air fresheners, and aerosols or sprays, including hair sprays (Addissie et al., 2021). Importantly, this study also showed a protective effect of folic acid intake during the first month of pregnancy (Addissie et al., 2021). Addissie et al. added several important features to their analysis that should lead the way for future epidemiological studies on HPE. First, controls included children with Williams-Beuren syndrome, a congenital anomaly with etiology and pathology distinct from HPE. This may help control for differences in recollection of exposures between parents of unaffected and affected children**.** Second, most probands underwent genetic testing for variants of *SHH*, *ZIC2*, *SIX3*, and *TGIF1*, allowing assessment of gene-environment interactions. Interestingly, the severity of HPE phenotypes in offspring of mothers with pathogenic variants was significantly *reduced* with higher amounts of maternal cheese consumption (Addissie et al., 2021). This could conceivably be related to the high cholesterol levels in cheese, as cholesterol is required for HH signaling (Radhakrishnan et al., 2020).

## Conclusion

**FIGURE 2:**
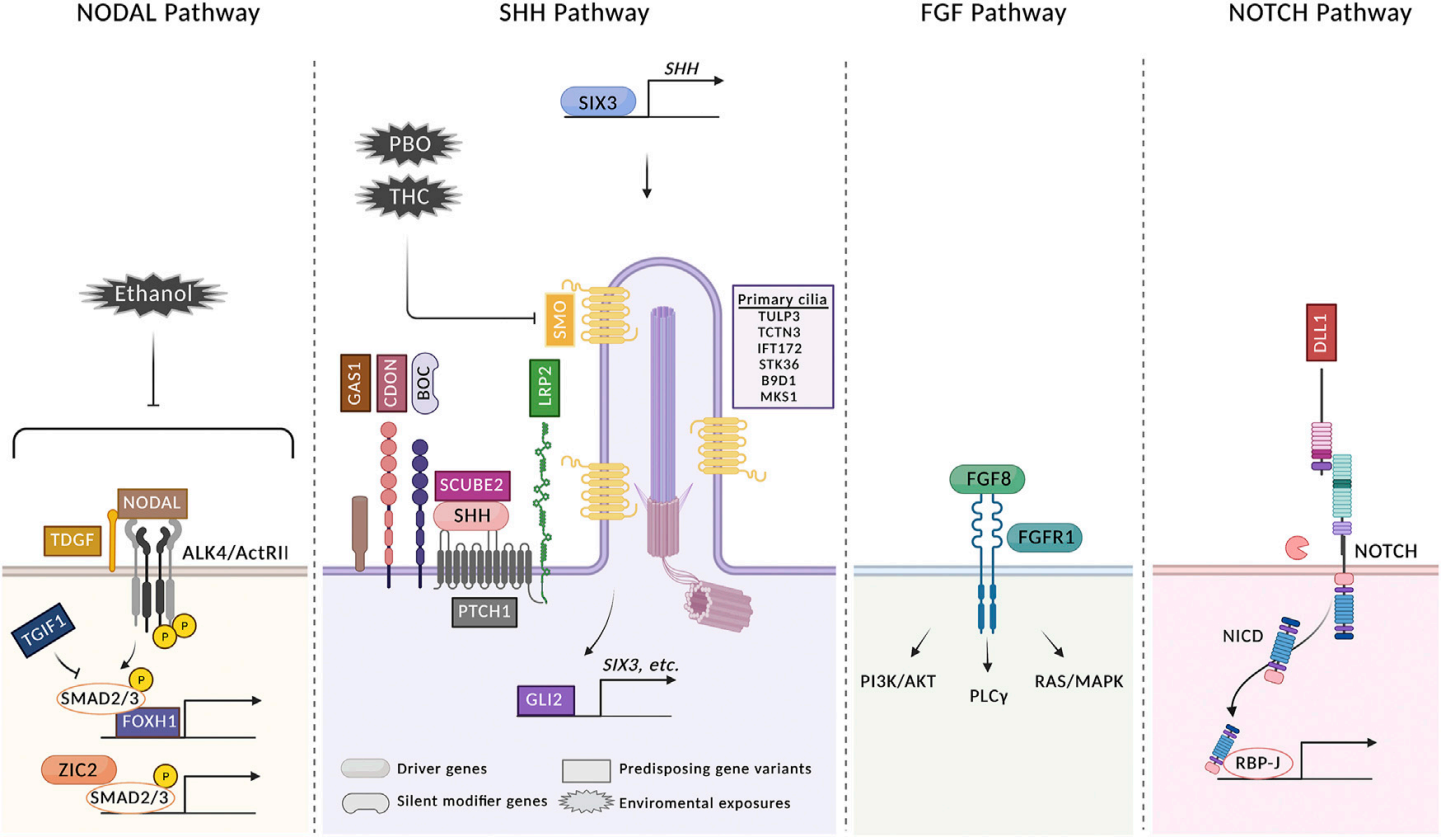
HPE arises from a confluence of multiple genetic and environmental risk factors. Four signaling pathways in which gene variants have been identified in HPE patients are shown. Variants in genes identified in HPE patients are classified as driver genes, silent modifier genes, and predisposing gene variants. Driver genes are defined as those accepted to be essential to the phenotype of the patient carrying a variant and include *SHH*, *ZIC2*, *SIX3*, *FGF8*, *and FGFR1*. A single silent modifier gene (*BOC*) is listed; see text for further discussion. All other genes are categorized as predisposing gene variants. Variants of these genes may function as drivers in individual HPE cases, but the relative infrequency of their involvement currently makes this difficult to assess, as it is also possible that they can function as modifiers of a more critical insult, genetic or environmental. Variants in some additional genes identified in HPE cases are not shown because their roles in these pathways are not known [see (Roessler et al., 2018b; Tekendo-Ngongang et al., 2020) for complete lists]. Three environmental risk factors are shown. PBO and THC both directly inhibit SMO. Ethanol inhibits Nodal signaling, but the direct target is not known. For simplicity, not every regulator of each pathway is pictured. See text for further details. The figure was created with BioRender.com.

HPE is almost certainly caused by a complex set of genetic and environmental risk factors (Figure 2). These factors interact with each other to affect the strength and duration of key developmental signaling pathways, thereby increasing the possibility that they fail to achieve the thresholds required for normal patterning. The same is likely true of many common birth defects, including congenital heart defects, neural tube defects, and oro-facial clefting (Krauss and Hong, 2016; Beames and Lipinski, 2020; Martinelli et al., 2020; Finnell et al., 2021; Kodo et al., 2021). Genome sequencing analyses and epidemiology, plus mechanistic studies with animal models, have provided conceptual insights into HPE etiology which should prove applicable to these other developmental disorders.

To fully understand HPE etiology, it is necessary to continue these efforts. Eventually, whole genome sequencing of trios will need to be performed to get a complete picture of the genetic contribution in individual cases. Variants in genes not previously associated with HPE were identified in the first rounds of WES, and more are likely to come. Furthermore, reproducible co-occurrence of variants in specific combinations of genes is hinted at by Kim et al. (2018). As these become clearer, it should shed light on mechanisms whereby incomplete deficiency of multiple pathways synergize to result in clinical phenotypes. Finally, it is known that mutations in transcriptional regulatory elements can occur in HPE (e.g., in a brain-specific enhancer for SHH expression) (Jeong et al., 2008), but the frequency of such events is unknown.

Assessment of gene-environment interactions in human studies will be very important as investigation of HPE and other developmental disorders with complex etiology progresses. Potential mechanisms of gene-environment interactions are myriad (Krauss and Hong, 2016); molecular insight into such mechanisms will be best addressed with animal models and *in vitro* systems. Interactions between non-genetic risk factors must also eventually be included. Animal models will be helpful here. A recent study showed that PAE and PBO synergized in a zebrafish model of craniofacial defects, some of which resemble HPE; moreover, this combination of environmental risk factors further interacted with heterozygous mutation of *shh* (Everson et al., 2020). It should be emphasized that many potential environmental risk factors may *require* complementary insults for their effects to manifest [e.g., THC in 129S6 mice (Lo et al., 2021)]. Additionally, the doses of potential teratogens that are, on their own, sufficient to produce phenotypes in animal models may not be achieved in average human populations (although they may occur in occupational settings or through excessive self-exposure, e.g., binge drinking). Subthreshold doses of such factors may be additive or synergistic in human populations and they may also interact with predisposing genetic sensitivities. Animal model studies are well positioned to illuminate such interactions, and may spur investigation of specific interactions in human populations. In summary, the study of HPE has produced important insights not only into how this complex and very common developmental disorder occurs but also concepts expected to shed light on causation in other similarly complex and frequently occurring birth defects.
